# Address of Dr. J. B. Patrick before the Southern Dental Association

**Published:** 1890-09

**Authors:** 


### Address of Dr. J. B. Patrick Before the Southern Dental Association, Atlanta, July 10th, 1890.

*Mr. Chairman and my professional brethren of Atlanta:*—Were it possible for me to imitate the grace, or rival the aptness of speech with which your welcome to Atlanta has been so cordially extended, I should feel no hesitation in occupying the enviable position which permits me, in behalf of my colleagues of the Southren Dental Association, to return you our hearty thanks for this hospitable greeting.

Words were unnecessary to inform us that when we came to the grand old State of Georgia we should find the latch-string hanging out and meet a multitude of the brave, self-reliant and inspiring men who rescued their city from the ruins and made it the cynosure of every commercial eye in the United States.

In the course of our wanderings as an association—from Maryland to Texas, wherever indeed, we have planted our tent poles—we have received a wealth of welcome. But here, where fire and the sword once laid waste your homes and business places, you furnish us in addition to your good fellowship with a type of the growth that may be achieved by high and earnest endeavor. By your example as citizens you teach us as dentists, the lesson that substantial progress is only to be attained by laboring, not for the benefit of the few, but for the good of all, and we shall not be slow to profit by it.

Our profession especially is one that should hide from view no

‡London Lancet, Jan. 25, 1890.

secret which concerns the welfare of humanity, and we are here in a true spirit of scientific inquiry, to show our hands, to exchange our experiences, and to impart to our brethren such knowledge as we may have acquired during the year in the laboratory and the clinique. New phases of observation and progress will doubtless be presented, and we hope to be able to demonstrate that the dentists of America have moved with quick step and solid column to the front, so that today, even in our youth, the Old World is adopting our ideas, and in the superiority of our scientific treatment and mechanical skill we everywhere stand unchallenged.

Permit me here, Mr. President, speaking for my Southern associates, to join you in a special welcome to those of our Northern brethren who may be present during the sessions of this convention. For several years after the war, owing to the fact that our dollars were too weak and inefficient to travel far from home and were always lonely in strange company, it was not practicable for many of our members, especially those from the extreme South and West, to attend the meetings of either of the two associations then in existence at the North, but we have had frequent occasion to meet our friends since, both under their own vine and fig tree and here in the home of the pine and palm. We have enjoyed their contributions to the literature of the Southern Dental Association and crossed weapons with them in argumentative strife, without other bloodshed, however, than that of some accommodating patient, and we are glad to see them here again. I hope they are prepared to open their usual store of rich experience and bring us new revelations of the possibilities of our profession.

Dentistry may be said to have started from the bare ground a little over fifty years ago. The practitioner felt grandly equipped if he possessed a turnkey, a half dozen drills, as many excavators and fillers, a few commonplace drugs and an ordinary easy chair with a head-rest. Yet the work then conscientiously done, even with these rude materials, would bear favorable comparison in point of durability, if not of beauty, with much that is performed by more scientific methods at the present time.

But what marvellous changes have since taken place! Now everything is prepared ready for use. The inventive and operative faculty of the American people, stimulated to its highest accomplishment, has produced appliances and originated methods to which Europe with all its scientific lore was long a stranger. The travelling tooth-puller, or, as he was facetiously called, the "gum carpenter," has given way to the college graduate, who, with his luxurious operating chair, dental engine, hand, automatic and electric mallets, duct compressors, rubber dam, pain obtunders, the different forms of gold, plastic materials and the thousands of devices to remedy defect, or relieve disease, as much represents the splendid advance of the dental profession as the reaping machine, the electric motor or the telephone in their respective departments of usefulness, mark the superiority of the present over any other age in the world's history.

It may be safely asserted that this progress is due to dental literature and dental colleges and conventions; in other words, to the interchange and suggestiveness of thought. When the *American Journal of Dental Science* appeared in 1839, the professional horizon was shrouded in darkness. Since then more than one hundred periodicals have been established, and it goes without saying that they have become potent factors in the collation and dissemination of all attainable knowledge concerning the improved mode of practice in the profession.

The first American society of dental surgeons was organized in 1840, closing its career sixteen years afterward. The Virginia State Society was founded in 1842, the Mississippi Valley Society in 1844, the Pennsylvania Society in 1845, and the New York State Society in 1847. All of these together with useful auxiliaries are still exerting direct influence upon our work. The first assemblage of the American Dental Convention took place in 1855. This was followed in 1859 by the American Dental Association. Our Southern Dental Association was organized just after the war. It embraces the field between Maryland and Texas, and it is not too much to say that it is one of the most progressive bodies of its kind on this Continent, for its aims and interests are universal.

Its members, of whom there are several hundred, are united in their work and wedded to a spirit of scientific inquiry, having for its object "the greatest good to the greatest number." We are not only endeavoring to maintain the high standard to which I have referred, but to elevate it to that altitude where it shall be regarded not as a mere specialty of one, but a peer among all the learned professions.

It is with a species of local pride that I say it, but the prestige of American dentistry in Europe was established by a former resident of Charleston, my own home, and I am informed that at the present time there is scarcely a city of considerable dimensions on the Continent in which are not to be found American practitioners, who following his example, have, so to speak, "set the pace" of scientific endeavor for their European cousins.

It is a source of national pride also that the first institution in the history of the world for special instruction in dentistry—the College of Dental Surgery, founded in Baltimore in 1840—served as a model for the university of Leipsic and Berlin, and has given a stimulus to the profession in the Old World which it did not before possess.

Now there are in this country twenty-two recognized dental colleges, prominent among which is your own in Atlanta, an institution of which the South may be justly proud—all engaged in the work of imparting the fundamental knowledge of the medical, surgical and mechanical principles that underlie the whole of dental science.

In addition to these are the special chairs for oral surgery and operative and mechanical dentistry established in a number of universities notably those of Michigan, Maryland, Pennsylvania the Vanderbilt and the University of Tennessee. Thus a higher and broader standard of education has been raised, a more thorough preliminary preparation has become necessary, and a course of instruction been presented that fully embraces the field of intellectuality in which the student may be best fitted for a professional career.

While our Association had its birth and was cradled in a dark hour, like our own beloved South, it has steadily grown strong and prosperous. During a part of our career, the wishes of some among us so far prevailed as to induce the adoption of a new title, that of the National Association, but old memories and old affections rushed back upon us as vivid as the time when they were our daily talk, and wedded as we were to these, for they always recalled our former struggles and triumphs, a year later witnessed our return to the old name. It is our birthright and we love it.

Of the advantages derived from our social intercourse in these annual meetings how much might be said! Ours is a broad field upon which men may meet from every section of the United States, and in their contact understand and appreciate each other far better than ever before. It is an arena in which thought is moulded and character is studied. It is a realm of opinion, a crucible of ideas, a school and a theatre; at once the spur and crown of ambition, as well as a tribunal which unmasks pretension and stamps real merit.

Employing a well-known simile, we are like a bundle of sticks laid together, whereof one kindles another. Not only are binding friendships thus formed, but in our clinics, where are witnessed various methods of practice, we have an opportunity of testing our own experience, criticising that of others, acquiring fresh ideas and gaining larger benefits than often accrue from the best written article in the books.

As Washington Irving has said in one of his essays, "the eye may be delighted with the smiling verdure of a lawn where every roughness is smoothed and every bramble eradicated, but he who would study nature in its wildness and variety must plunge into the forest, explore the glen, stem the torrent and dare the precipice."

It should matter little to us whether dentistry is a specialty of medicine or a distinct and independent profession, for if either proposition or both were established it would not change the fact that in our alliance with the sciences and arts we represent much of the most useful knowledge of Christendom. The true dentist is not only a mechanic, an inventor and builder, often from original designs, but in his ranks are found anatomists, physiologists, pathologists, chemists, microscopists, botanists, geologists and metallurgists, and according to his skill in the application of this diversified knowledge will be the measure of good he may confer upon mankind.

Naturally it is a source of gratification that when the International Medical Congress assembled in Washington, three years ago, a dental section was organized for the discussion of subjects pertaining to our profession, but beyond the mere fact that dentistry was for the first time officially recognized as a legitimate branch of the healing art, it added nothing to the status we have occupied for nearly half a century, or since the graduates of our dental colleges began to be sought in respectful consultation by physicians who stand in far greater need of a dental education than dentists of a medical course—as there are many men who make good doctors, but it is not always the best of doctors who will make a good dentist, and in order to be a good dentists it is not necessary to be able to superintend the birth of a baby.

There are many things in a medical college which it is indispensable that a dentist should know in order that he may be qualified for the practice of his profession, but he may learn them elsewhere, and the title of M.D. is not a necessary addendum to that which has been honorably acquired by a graduate of a dental college.

I believe it to be true of all professions, especially our own, that what we know most, that which we apply and communicate as the result of practical study, is acquired after we have left our instructors. No college can make a truly scientific man, and the time of graduation is but the commencement of another system of education. Many are the practitioners of the day, especially among the old men, who, having been compelled by circumstances to enter the profession of dentistry as it were by the back door, have subsequently by their own individual efforts in scientific study, in the laboratory, workshop and operating room, achieved the highest results and reaped the most valuable rewards.

But I have already spoken too long. We are impatient to begin our deliberations and listen to the papers and discussions that are to tell us of the progress made since we last assembled.

We shall doubtless exchange many opinions and experiences, perhaps on some points "agree to disagree," as Shakespeare says, "striving to better, oft we mar what's well," but when our labors are ended we shall return to our homes filled with new endeavor and prouder than ever before of that calling of which Prof. Oliver Wendell Holmes once wrote:

"It has established and prolonged the reign of beauty, it has added to the charms of social intercourse and lent perfection to the accents of eloquence; it has taken from old age its most unwelcome feature and lengthened the enjoyment of human life far beyond the limit of the years when the toothless patriarch of old exclaimed: 'I have no pleasure in them.'"

